# Non-targeted urine metabolomics and associations with prevalent and incident type 2 diabetes

**DOI:** 10.1038/s41598-020-72456-y

**Published:** 2020-10-05

**Authors:** Samira Salihovic, Corey D. Broeckling, Andrea Ganna, Jessica E. Prenni, Johan Sundström, Christian Berne, Lars Lind, Erik Ingelsson, Tove Fall, Johan Ärnlöv, Christoph Nowak

**Affiliations:** 1 School of Medical Sciences, Örebro University, Örebro, Sweden; 2 Department of Medical Sciences, Molecular Epidemiology and Science for Life Laboratory, Uppsala University, Uppsala, Sweden; 3 Analytical Resources Core: Bioanalysis and Omics Center, Colorado State University, Fort Collins, CO USA; 4 Institute of Molecular Medicine Finland, University of Helsinki, Helsinki, Finland; 5 Analytic and Translational Genetics Unit, Massachusetts General Hospital, Boston, MA USA; 6 Stanley Center for Psychiatric Research, Broad Institute of MIT and Harvard, Cambridge, MA USA; 7 Department of Horticulture and Landscape Architecture, Colorado State University, Fort Collins, CO USA; 8 Department of Medical Sciences, Clinical Epidemiology, Uppsala University, Uppsala, Sweden; 9 The George Institute for Global Health, University of New South Wales, Sydney, Australia; 10 Division of Cardiovascular Medicine, Department of Medicine, Stanford University School of Medicine, Stanford, CA USA; 11 Stanford Cardiovascular Institute, Stanford University, Stanford, CA USA; 12 School of Health and Social Studies, Dalarna University, Falun, Sweden; 13 Department of Neurobiology, Care Sciences and Society (NVS), Family Medicine and Primary Care Unit, Karolinska Institutet, Huddinge, Sweden

**Keywords:** Metabolomics, Biomarkers, Endocrinology, Endocrine system and metabolic diseases, Metabolic disorders

## Abstract

Better risk prediction and new molecular targets are key priorities in type 2 diabetes (T2D) research. Little is known about the role of the urine metabolome in predicting the risk of T2D. We aimed to use non-targeted urine metabolomics to discover biomarkers and improve risk prediction for T2D. Urine samples from two community cohorts of 1,424 adults were analyzed by ultra-performance liquid chromatography/mass spectrometry (UPLC-MS). In a discovery/replication design, three out of 62 annotated metabolites were associated with prevalent T2D, notably lower urine levels of 3-hydroxyundecanoyl-carnitine. In participants without diabetes at baseline, LASSO regression in the training set selected six metabolites that improved prediction of T2D beyond established risk factors risk over up to 12 years' follow-up in the test sample, from C-statistic 0.866 to 0.892. Our results in one of the largest non-targeted urinary metabolomics study to date demonstrate the role of the urine metabolome in identifying at-risk persons for T2D and suggest urine 3-hydroxyundecanoyl-carnitine as a biomarker candidate.

## Introduction

Type 2 diabetes mellitus (T2D) is a metabolic disease characterized by raised fasting glucose levels due to insulin resistance and impaired insulin production. It is a leading cause of cardiovascular disease, blindness and kidney failure^1^. The 2017 global estimate of 425 million persons with diabetes is projected to increase by 48% to 629 million by 2045^2^. A continuing challenge is the identification of persons at high risk of T2D, particularly in the absence of established risk factors such as obesity and poor diet^3,4^. This need is underscored by a 2017 survey by the charity *Diabetes UK*, where a top research priority for persons affected by T2D was to "identify people at high risk of type 2 diabetes and help to prevent the condition from developing"^5^. Another challenge has been the identification of currently unknown molecular mechanisms of T2D that could act as novel treatment targets^6^.

Non-targeted (or untargeted) metabolomics describes the assessment of small molecules (< 1,500 Daltons in molecular weight) in biological specimens and comprises a broad range of peptides, carbohydrates, lipids and nucleic acids. Non-targeted methods such as ultra-performance liquid chromatography coupled to quadrupole-time-of-flight mass spectrometry (UPLC-QTOFMS) capture all metabolite signals detectable by the method at hand without *a priori* selection. In an electrospray ionization source (ESI), effluents from the liquid chromatography system are nebulized at atmospheric pressure and ionization occurs through the application of a strong electric field on the surface of the effluent droplets as they elute from the nebulizer. The size of the charged droplets diminishes as the formed molecular ions and molecular adducts travel towards the mass spectrometer for analysis, collision induced dissociation, and mass detection. The accurate mass, mass spectra, and retention time of each molecular ion is matched to metabolites by comparison to internal and external standards or public databases^7–9^. Serum and plasma metabolomics have been used to discover biomarkers and improve risk prediction for insulin resistance and T2D^10–13^. Far less attention has been paid to the urinary metabolome. A genome-wide association study showed about a two-thirds overlap between urinary and plasma metabolite loci in the genome^14,15^. One study in ~ 3,900 healthy persons found correlations between five-year change in glycated hemoglobin levels and baseline levels of urinary metabolites such a betaine and trimethylamine^16^. A cross-sectional study reported 94 metabolites in plasma, urine or saliva samples that differed between persons with and without T2D^17^. We are unaware of any published study that uses large-scale non-targeted urinary metabolomics for biomarker discovery or risk prediction of incident T2D.

Here, we use non-targeted UPLC-MS urinary metabolomics in two community-based cohorts of > 1,400 Swedish adults to discover metabolites associated with prevalent T2D and to assess whether urinary metabolomics improves risk prediction of incident T2D beyond an established clinical risk score.

## Results

**Figure 1:**
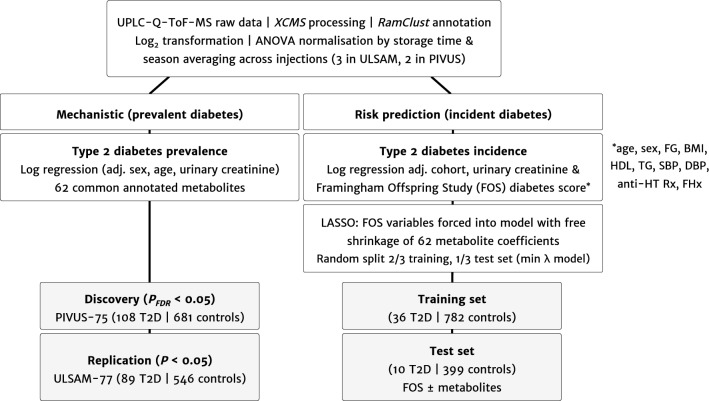
Study design.

We included 789 participants of the PIVUS study (108 prevalent cases of T2D) and 635 participants of the ULSAM study (89 cases of prevalent T2D). Figure 1 shows the study flow, and baseline characteristics are displayed in Table 1.

**Table 1:** Participant characteristics.

	PIVUS	ULSAM
Number of persons with/without prevalent T2D	108/681	89/546
Women, n (%)	404 (51.1%)	0 (0%)
Age, years	75.3 (0.2)	77.5 (0.8)
BMI, kg/m^2^	26.8 (4.4)	26.3 (3.4)
Fasting glucose, mmol/l	5.8 (1.6)	5.9 (1.4)
Systolic BP, mmHg	148 (22)	151 (10)
Diastolic BP, mmHg	88 (11)	81 (10)
Total cholesterol, mmol/l	5.4 (1.1)	5.4 (1.0)
Triglycerides, mmol/l	1.4 (0.7)	1.4 (0.7)
LDL cholesterol, mmol/l	3.4 (1.0)	3.5 (0.9)
HDL cholesterol, mmol/l	1.5 (0.5)	1.3 (0.3)
Current smoker, n (%)	48 (6%)	47 (7.4%)
Family history diabetes, n (%)	136 (17.2%)	83 (13.1%)
Antihypertensive medication, n (%)	358 (45.4%)	258 (40.6%)
Lipid medication, n (%)	210 (26.7%)	115 (18.1)

In the discovery sample PIVUS, 7 out of 62 preliminarily annotated metabolites measured in both cohorts were associated with prevalent T2D after adjustment for sex, age and urinary creatinine at a false discovery rate (FDR) < 0.05: 3-methyxanthine (odds ratio, OR, per standard deviation increase and 95% confidence interval, CI, 0.70 [0.59, 0.84], *P* = 7.93 × 10^−5^), 2-hepteneoglycine (OR, 0.70, 95% CI [0.58, 0.84], *P* = 1.31 × 10^−4^), nonanoylcarnitne (OR, 0.71, 95% CI [0.57, 0.88], *P* = 1.44 × 10^−3^), L-tyrosine (OR, 1.36, 95% CI [1.12, 1.66], *P* = 2.05 × 10^−3^), irinotecan metabolite NPC (OR, 0.76, 95% CI [0.63, 0.91], *P* = 3.53 × 10^−3^), 3-hydroxyundecanoyl-carnitine (OR, 0.78 95% CI [0.65, 0.93], *P* = 4.71 × 10^−3^) and vildaglipitin (OR, 0.79, 95% CI [0.67, 0.93], *P* = 5.47 × 10^−3^) (Table 2).

**Table 2:** Associations between urinary metabolite levels and prevalent T2D in the discovery and replication samples in logistic regression adjusted for age, sex (for PIVUS only) and urinary creatinine per standard deviation unit increase in metabolite level.

Discovery (PIVUS, n = 789)			Replication (ULSAM, n = 635)		
Metabolite (preliminary annotation with RamClust)	OR (95% CI)	*P* value		OR (95% CI)	*P* value
3-Methylxanthine	0.70 (0.59–0.84)	7.93 × 10^−5^	3-Methylxanthine	0.84 (0.68–1.05)	0.125
2-Hepteneoylglycine	0.70 (0.58–0.84)	1.31 × 10^−4^	2-Hepteneoylglycine	1.06 (0.82–1.36)	0.681
Nonanoylcarnitine	0.71 (0.57–0.88)	1.44 × 10^−3^	Nonanoylcarnitine	0.71 (0.56–0.89)	3.11 × 10^−3^
L-Tyrosine	1.36 (1.12–1.66)	2.05 × 10^−3^	L-Tyrosine	1.04 (0.81–1.32)	0.769
NPC (irinotecan metabolite)	0.76 (0.63–0.91)	3.53 × 10^−3^	NPC (irinocetan metabolite)	0.85 (0.68–1.06)	0.154
3-hydroxyundecanoyl-carnitine	0.78 (0.65–0.93)	4.71 × 10^−3^	3-hydroxyundecanoyl-carnitine (C-580)	0.61 (0.47–0.79)	1.56 × 10^−4^
Vildagliptin	0.79 (0.67–0.93)	5.47 × 10^−3^	Vildagliptin	0.93 (0.73–1.19)	0.572
Methysergide	0.77 (0.64–0.94)	9.49 × 10^−3^	–	–	–
Pilocarpine	0.78 (0.65–0.95)	0.012	–	–	–
2-Methoxyestradiol	0.79 (0.66–0.95)	0.012	–	–	–
Phthalic anhydride	1.26 (1.04–1.53)	0.016	–	–	–
(3a,5b,7a,12a)-24-[(carboxymethyl)amino]-1,12-dihydroxy-24-oxocholan-3-yl-b-D-Glucopyranosiduronic acid	1.24 (1.01–1.52)	0.044	–	–	–
Methylcysteine	0.85 (0.72–1.00)	0.047	–	–	–
L-Urobilin	1.22 (1.00–1.50)	0.053	–	–	–
Dihyroxy-1H-Indole-glucuronide	1.24 (0.99–1.55)	0.06	–	–	–
Sotalol	0.79 (0.62–1.02)	0.067	–	–	–
N-Stearoyl-phenylalanine	1.20 (0.98–1.46)	0.073	–	–	–
Glutarylcarnitine	0.85 (0.70–1.02)	0.078	–	–	–
3-hydroxydecanoyl-carnitine (C-580)	0.85 (0.71–1.02)	0.087	–	–	–
2-Octenoylcarnitine	0.87 (0.73–1.02)	0.089	–	–	–
Dynorphin-A	1.21 (0.97–1.50)	0.089	–	–	–
trans-Ferulic acid	1.19 (0.97–1.45)	0.091	–	–	–
3-hydroxy-2-methyl-2-sulfooxy-methyl-propanoic acid	0.84 (0.68–1.03)	0.091	–	–	–
C4H8O2S4	0.85 (0.71–1.03)	0.093	–	–	–
Glutaconylcarnitine	1.20 (0.96–1.50)	0.102	–	–	–
Proline-betaine	0.85 (0.70–1.04)	0.117	–	–	–
Codeine-6-glucuronide	0.80 (0.60–1.06)	0.119	–	–	–
C5H14S	1.18 (0.96–1.47)	0.123	–	–	–
4-Hepteneoylglycine	0.85 (0.69–1.05)	0.124	–	–	–
Hydralazine-pyruvate-hydrazone	0.88 (0.72–1.07)	0.193	–	–	–
Estrone	0.89 (0.75–1.06)	0.194	–	–	–
L-Proline	1.14 (0.93–1.40)	0.2	–	–	–
Cortexolone	0.89 (0.74–1.07)	0.207	–	–	–
Prednicarbate	1.14 (0.92–1.41)	0.238	–	–	–
Pantothenic acid	1.13 (0.92–1.38)	0.245	–	–	–
5-3-4-Dihydroxyphenyl-gamma-valerolactone-3-O-methyl-4-O-glucuronide	0.91 (0.74–1.11)	0.352	–	–	–
Androstenedione	0.92 (0.76–1.12)	0.399	–	–	–
gamma-Glutamylphenylalanine	1.09 (0.89–1.34)	0.407	–	–	–
Chenodeoxycholic acid-glycine conjugate	1.09 (0.88–1.34)	0.43	–	–	–
N-acetyltryptophan	1.08 (0.88–1.34)	0.45	–	–	–
C4NO3P	0.92 (0.74–1.14)	0.454	–	–	–
2-Hexenoylcarnitine	0.93 (0.76–1.14)	0.495	–	–	–
Glucosamine	1.07 (0.87–1.33)	0.51	–	–	–
gamma-Glutamylvaline	1.06 (0.86–1.31)	0.563	–	–	–
Pyroglutamic acid	0.95 (0.79–1.14)	0.581	–	–	–
Dihydroferulic acid-4-O-glucuronide	1.06 (0.87–1.29)	0.584	–	–	–
Nabilone	1.05 (0.86–1.29)	0.619	–	–	–
Acetaminophen glucuronide	0.96 (0.79–1.17)	0.662	–	–	–
Ajmaline	1.04 (0.85–1.28)	0.671	–	–	–
7-hydroxygranisetron	1.04 (0.84–1.29)	0.699	–	–	–
Indoleacrylic acid	1.0 4 (0.85–1.28)	0.714	–	–	–
Paramethadione	0.97 (0.79–1.18)	0.747	–	–	–
5-Hydroxy-6-methoxyindole-glucuronide	1.03 (0.84–1.26)	0.757	–	–	–
Tyrosinamide	0.97 (0.79–1.19)	0.772	–	–	–
Zolmitriptan	0.98 (0.80–1.20)	0.855	–	–	–
6b-hydroxybudesonide	1.01 (0.82–1.25)	0.893	–	–	–
Atenolol acid	0.99 (0.81–1.22)	0.919	–	–	–
Tranexamic acid	0.99 (0.81–1.21)	0.936	–	–	–
L-Glutamine	0.99 (0.81–1.22)	0.945	–	–	–
Hesperetin	1.00 (0.82–1.23)	0.985	–	–	–
L-Octanoylcarnitine	1.00 (0.81–1.24)	0.999	–	–	–
11-Hydroxyprogesterone-11-glucuronide	1.00 (0.81–1.24)	0.999	–	–	–

Metabolites associated at a false discovery rate of 5% in the discovery sample PIVUS were tested in ULSAM. The names reflect the initial, automated data-driven annotation as explained in the Methods section. The shown *P* values are unadjusted.

**Figure 2:**
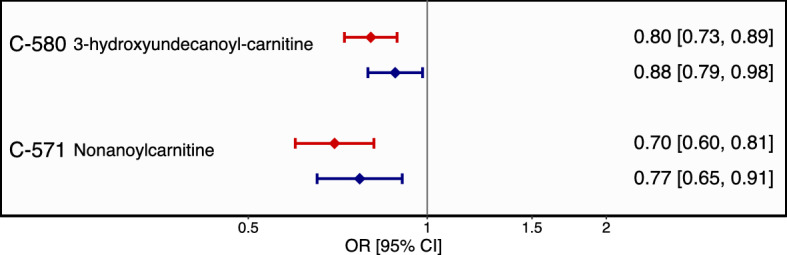
Associations of the replicated urinary metabolites and prevalent T2D in the combined sample (n = 1,424). Results from logistic regression adjusted for age, sex, cohort and urinary creatinine (red color) and with additional adjustment for BMI, HDL-cholesterol, triglycerides, systolic and diastolic blood pressure, hypertension and family history of diabetes (blue color). Error bars denote 95% CI around odds ratios per standard deviation increase in urinary metabolite level.

Two of these preliminarily annotated metabolites were associated with prevalent T2D in the replication sample ULSAM at the nominal significance level: 3-hydroxyundecanoyl-carnitine (OR, 0.61, 95% CI [0.47, 0.79], *P* = 1.56 × 10^−4^) and nonanoylcarnitine (OR, 0.71, 95% CI [0.56, 0.89], *P* = 3.11 × 10^−3^). In-depth manual annotation (Supplementary Text and Supplementary Figures 1–10) confirmed the annotation of 3-hydroxyundecanoyl-carnitine. The second compound was annotated as the sodium adduct of nonanoylcarnitine (Supplementary Figures 8–13). Figure 2 shows the associations of the two replicated metabolite features in the combined cohorts with and without additional adjustment for T2D risk factors in the Framingham Offspring Study (FOS) diabetes model.

**Figure 3:**
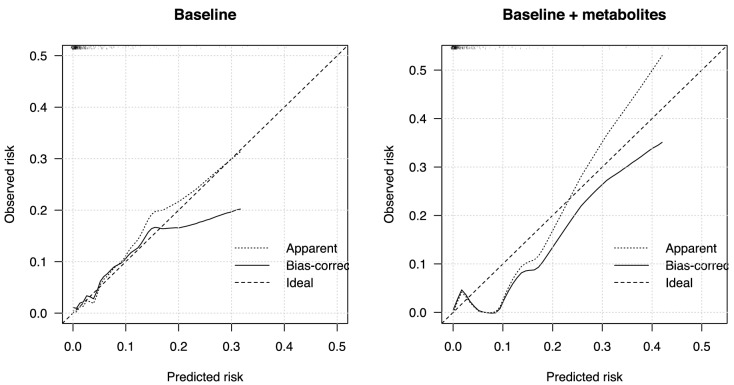
Calibration plots in the baseline FOS model (left panel) and the baseline-plus-metabolites model (right panel) in the test sample (n = 409).

To assess associations with incident T2D, we combined both cohorts after excluding prevalent cases of T2D at baseline (n = 1,227) and randomly split the sample into a two-thirds training (n = 818) and one-third test set (n = 409). Over a maximum of 12 years′ follow-up (mean 6.32 ± 3.1 years), there were 36 and 10 incident cases of T2D in the training and test sets, respectively. LASSO regression in the training set that forced cohort, age, sex, urinary creatinine and the FOS variables into the model selected six out of the 62 metabolites as the optimal parsimonious model to predict risk of T2D (C_5_H_14_S, indoleacrylic acid, sotalol, tranexamic acid, trans-ferulic acid, (3a,5b,7a,12a)-24-[(carboxymethyl)amino]-1,12-dihydroxy-24-oxocholan-3-yl-b-D-glucopyranosiduronic acid). In the holdout test set, the baseline model C statistic was 0.866 (95% CI, 0. 786–0.946, Nagelkerke's pseudo-R^2^ 0.271), and the baseline-plus-metabolite model C was 0.892 (95% CI, 0.812–0.972, pseudo-R^2^ 0.354; change in model fit likelihood ratio test, *P* = 0.276). Hosmer–Lemeshow test in the test sample did not reject the null hypothesis of good fit (baseline model *P* = 0.398, baseline-plus-metabolite model *P* = 0.257). In contrast, calibration plots of observed and predicted risk indicated that while the baseline model was well calibrated, the baseline-plus-metabolites model showed signs of underestimation of risk (Fig. 3). This discrepancy may be due to the small number of cases—particularly in the test set—which resulted in low statistical power of the formal calibration test and does not allow reliable conclusions about the merits of the model.

## Discussion

In 1,424 Swedish adults enrolled in two community-based cohorts, we discovered associations between prevalent T2D and lower urinary levels of 3-hydroxyundecanoyl-carnitine and the sodiated adduct of nonanoyl-carnitine (Supplementary Text). We also found indications for improved risk prediction for incident T2D over an average 6-year follow-up period after adding six urinary metabolites to an established diabetes risk score that did not, however, reach statistical significance. The small number of cases demands cautious interpretation of the prediction results for incident T2D.

### Association between urinary 3-hydroxyundecanoyl-carnitine level and T2D

3-hydroxyundecanoyl carnitine (C_18_H_35_NO_5_, HMDB0061637) belongs to the group of acylcarnitines, which are essential organic compounds composed of a fatty acid with a carboxylic acid attached to carnitine by an ester bond that are essential intermediates in fatty acid metabolism. This odd-numbered C11-carnitine occurs with relatively low abundance in the circulation and tissues when compared to even-chain acylcarnitines. The principal origin of odd-numbered medium-chain acylcarnitines remains elusive; odd-chain acylcarnitines originate both, from branched-chain amino acid catabolism, and to a lesser extent the peroxisomal processes of fatty acid alpha oxidation^18^. Despite the low abundance of odd-numbered acylcarnitines in biological matrices, the use of mass spectrometric methods has prompted the detection of C11-carnitine and other odd-numbered acylcarnitines in animal and human plasma^19–21^, urine^20,22^, as well as liver and kidney^23,24^. However, the distribution of odd-numbered acylcarnitines and other acylcarnitines between tissues, plasma, and renal excretion remains poorly understood^25^.

Whilst levels of various acylcarnitines in the circulation^18,19^ and urine^20^ have been associated with increased risk of T2D and insulin resistance^21^, there is a dearth of evidence linking specifically the odd-numbered medium-chain C11-carnitine to diabetes. In a comprehensive analysis including > 110 acylcarnitines in plasma and urine of leptin-deficient (*db/db*) mice, an accumulation of plasma medium- and long-chain acylcarnitines was accompanied by a decrease in urinary odd-numbered (C7, C9, and C11) medium-chain acylcarnitine levels^20^. None of the other seven acylcarnitines (variants of C5, C6, C8 and C10-carnitine) among the 62 automatically annotated metabolites in our study were statistically significantly associated with prevalent T2D (Supplementary Text).

Our study is the first in human participants to report an association between lower levels of urinary C11-carnitine and prevalent T2D. Lack of power to detect associations with other carnitine metabolites cannot be excluded, as our sample size was limited. Annotation certainty of this metabolite in our sample at Metabolomics Standards Initiative (MSI) confidence level 2 is comparatively good (Supplementary Text and Supplementary Figures 1–3), although our inability synthesize authentic standards for external validation of the annotation leaves some uncertainty.

### Associations between another urinary carnitine metabolite and T2D

Lower urinary levels of C-571 were associated with prevalent T2D both before and after additional adjustment for established T2D risk markers (Fig. 2). This metabolite was initially computationally annotated as N-jasmonoylisoleucine, but review of the spectral data strongly suggests this signal as the sodiated adduct of nonanoyl-carnitine with the association signal possibly due to a statistical artifact (Supplementary Text, Supplementary Figures 10–15). The precursor molecular ion (M + H) of nonanoyl-carnitine compound was not associated with any of the outcomes in our study and the signal for the sodiated adduct of nonanoylcarnitine could, in our opinion, be a statistical artefact. We are therefore unable to further explore the possible biology behind this association but provide detailed information on the annotation in the Supplementary Text and Supplementary Figures.

### Strengths and Limitations

We report the first epidemiological study of non-targeted urinary metabolomics to assess the risk of prevalent and incident T2D in two independent community-based cohorts. Strict statistical controls for multiple testing, a discovery/replication design, over 10 years of follow-up in the ULSAM cohort, and the unbiased non-targeted metabolomics method are strengths of our study. Limitations include the limited power for incident T2D analysis and annotation uncertainties. The ULSAM cohort included only men, whilst the PIVUS cohort had a balanced sex ratio (all analyses were adjusted for sex). Our study used deep-frozen urine samples collected several years before the UPLC-MS technology became available, necessitating analysis of spot urine samples in PIVUS and 24-h urine collections in ULSAM. Analyses were adjusted for type of sample collection and difference in urine concentration (using creatinine levels as a proxy), but the different sampling methods may have impacted the results. In the absence of external validation and reanalysis of the samples (that were used up in the analysis), our annotation of metabolites remains unconfirmed. Future studies should strive for more controlled settings with regards to the collection of urine samples.

### Conclusion

In our metabolomics study in over 1,400 adults, lower urinary levels of 3-hydroundecanoyl-carnitine were associated with prevalent T2D. We were unable to assign molecular identities to another T2D-associated signal, but provide extensive discussion of the mass spectral characteristics and possible identities. We report our complete results despite remaining annotation uncertainties as a pioneering effort to study non-targeted urinary metabolomics and T2D without *a priori* selection of potential metabolites or biomarkers of interest, and as our explanations of the analytical pipeline makes an innovative and informative contribution to the field of human metabolism research. The field of non-targeted metabolomics is young and the growing availability of comparison structures in molecular databases will improve the identification of metabolites in the future.

## Methods

### Participants

#### Uppsala Longitudinal Study of Adult Men (ULSAM)

Between 1970–1973, ULSAM enrolled 2,322 (81.7%) of all 2,841 men born between 1920–1924 who were residents of Uppsala county, Sweden^26^. Regular biomedical assessments have been carried out ever since as detailed here (https://www.pubcare.uu.se/ulsam/). The current study used data and a 24-h urine collection at age 77 years. Participants were followed up until assessment at 93 years of age or death according to the Swedish Death register. Urine metabolomics data from 635 individuals out of 839 that attended assessment were available (missing individuals are due to missing urine samples or insufficient sample quality as metabolomics was carried out in the 2010s on biobank samples obtained at assessment age 77 years in the early 1990ies).

#### Prospective Investigation of the Vasculature in Uppsala Seniors (PIVUS)

In 2001, the PIVUS study (https://www.medsci.uu.se/pivus/) enrolled 50% (n = 1,016) of a random sample of Uppsala community residents aged 70 years with the aim of comparing different measures of arterial compliance^27^. The current study is based on the assessment at age 75 years where spot urine sample were collected and participants were followed until re-assessment at 80 years of age or death. We included urine metabolomics data from 789 participants who had deep-frozen urine samples of sufficient quality available at the point of analysis in the 2010s.

### Non-targeted metabolomics

Non-targeted metabolomics profiling of urine samples was carried out by ultra-performance liquid chromatography (UPLC) on a Waters Acquity UPLC system coupled to a Waters Xevo G2-Time-Of-Flight-Mass Spectrometry (TOFMS) platform at Colorado State University (Fort Collins, CO, USA). Data acquisition in the positive electrospray ion mode with a mass-to-charge ratio (m/z) range of 50–1,200 at 5 Hz was alternately performed at collision energies of 6 V and 15–30 V without discrimination or pre-selection. Cohorts were analyzed independently in multiple batches. Every set of 20 authentic samples was analyzed in triplicates interspersed with procedural blanks and control samples. There were 3,352 injection samples in ULSAM and 3,158 samples in PIVUS. Following data processing in *XCMS*^28^, there were 4,406 features in ULSAM and 3,615 features in PIVUS (including the control samples). LOESS curve normalization to correct for shift in intensity over time and probabilistic quotient normalization (PQN) to normalize samples based on dilution factors obtained from the median intensity across samples were carried out. *XCMS* parameters used for peak detection were nSlaves = 200, method = centWave, ppm = 25, peakwidth = c(2:25), snthresh = 11, mzCenterFun = wMean, integrate = 2, mzdiff = 0.01, prefilter = c(1,100). Peak alignment by retention time was carried out with the *obiwarp* method. Quality control included manual inspection of plots of total ion counts per sample and plots of peak number by retention time. Peaks were grouped with the parameters bw = 2, minfrac = 0.10, max = 1,000, mzwid = 0.01, sleep = 0.0001. Features with correlations across triplicate injections below 0.2 were removed; the triplicate peaks of features passing quality control were averaged. The final number of features was 4,084 in ULSAM and 3,178 in PIVUS. *RAMClustR* (version 1.0.4)^29^ was used to cluster features into spectra, *interpretMSSpectrum*^30^ was used to infer the molecular ion, and *MS-Finder*^31^ was used to annotate metabolites. Only annotated, quality-controlled metabolite features measured in both PIVUS and ULSAM were included in this study. Because this data-driven non-manual annotation can be liable to statistical artefacts, we refer to it as "preliminary/initial annotation" in the text. For all outcome-associated features, we went back to the original UPLC-MS data and carried out manual in-depth review to verify or refute the preliminary annotation. We present these validation steps for the main results of this study in the Supplementary Text and Supplementary Figures.

### Outcome definition

In ULSAM, diabetes was defined as fasting plasma glucose ≥ 7 mmol/L, glycated hemoglobin HbA1c ≥ 6.5% (48 mmol/mol), use of anti-diabetic medication according to the Swedish Prescribed Drug Register ATC code A10, and/or diagnosis of T2D according to the National Patient Register. In PIVUS, diabetes was defined as fasting plasma glucose concentration ≥ 7 mmol/L, use of anti-diabetic medication, and/or diagnosis of T2D according to validated hospital records (whole blood glucose values were transformed to plasma concentrations by adding 11%). HbA1c measurements were not available in PIVUS. More information on the cohorts and assessments are available here https://www.pubcare.uu.se/ulsam/ (ULSAM), and here https://www.medsci.uu.se/pivus/ (PIVUS).s

### Statistical analysis

For association analyses with T2D, we included all 62 preliminarily annotated metabolites present in both cohorts and excluded all features that could not be annotated or were present in only one of the cohorts. Log_2_-transformed metabolite signals were adjusted by ANOVA-type normalization within each injection run for winter season (an indicator variable for sampling during November to March) and storage time between sampling and UPLC-MS analysis, followed by averaging across injections.

We divided the association analysis into two parts (Fig. 1): In part 1, we used logistic regression adjusted for age, sex and urinary creatinine (measured with a colorimetric assay IL Test Creatinine 181672–00 on a Monarch 2000 centrifugal analyzer [Instrumentation Laboratories, Lexington, MA, USA] in ULSAM; and with a modified Jaffe reaction on an Architext Ci8200 analyzer [Reagent 3L81, Abbot, Abbot Park, IL, USA] in PIVUS) to test associations between each urinary metabolite (scaled to standard deviation units) and prevalent T2D at baseline. Urinary creatinine was included as a covariate because it was strongly associated with the dominant principle components in principle component analysis (implemented as part of the *XCMS* normalization steps), and to control for between-sample variation in urine concentration and sampling method (24 h versus spot collection). Metabolites associated at a false discovery rate (FDR) < 0.05 in the discovery sample PIVUS were tested in the replication sample ULSAM. In part 2, we used LASSO L1-regularised logistic regression to select urinary metabolites that together improved risk prediction for incident T2D when added to the risk factors in the Framingham Offspring Study (FOS) diabetes risk score^32^. We combined both cohorts, excluded all cases of prevalent T2D at baseline and randomly split the dataset into a 2/3 training and 1/3 holdout test set. The training dataset was used to develop the LASSO model by tenfold bootstrapped internal cross-validation and the test set was used only once to evaluate performance of the selected model with regard to risk discrimination (C statistic), calibration (plots of observed against predicted risk), goodness-of-fit (Hosmer–Lemeshow test) and explained variance (Nagelkerke's pseudo-R^2^). To develop the model in the training set, we forced cohort status and the FOS variables (age, sex, parental history of diabetes, body mass index, blood pressure, fasting glucose, HDL-cholesterol, triglycerides), into the model and allowed free shrinkage on all 6 urinary metabolite regression coefficients. Analyses were carried out in R version 3.3.3.

### Study approval

All participants provided written informed consent. The study was approved by the Regional Ethical Review Board of Uppsala University (Dnr. 251/90; 97/329; 2/605 and 2007/338 for ULSAM; Dnr. 00,419; 2005/M-079 and 2011/045 for PIVUS) and has been carried out in accordance with the principles of the Declaration of Helsinki as revised in 2008. Data handling since May 2018 has been in accordance with the EU protection regulation 2016/679 ("GDPR").

### Data and resource availability

Individual level data from ULSAM and PIVUS are not deposited in the public domain, as existing ethical permits and Swedish/EU data protection regulations do not allow this. Full datasets are made available to researchers who meet the criteria for confidential data access as stipulated by participant informed consent and institutional review board/ethics committee permission at Uppsala University (Uppsala, Sweden). Data access in ULSAM is granted through the Interdisciplinary Collaboration Team on Uppsala Longitudinal Studies (ICTUS; https://www2.pubcare.uu.se/ULSAM/res/proposal.htm; contact: vilmantas.giedraitis@pubcare.uu.se). Data from the PIVUS study can be applied for at the PIVUS steering committee (https://www.medsci.uu.se/pivus/; contact: lars.lind@medsci.uu.se).

De-identified raw mass spectrometry data (without phenotype or other identifying information) and the analysis code can be obtained without prior ethical or legal approval from the main author (christoph.nowak@ki.se).

## Supplementary information

Supplementary file1

## Data Availability

The authors report that, for approved reasons, some access restrictions apply to the data in this study. Individual level data from ULSAM and PIVUS are not deposited in the public domain, as existing ethical permits do not allow this. Full datasets are made available to researchers who meet the criteria for confidential data access as stipulated by participant informed consent and institutional review board/ethics committee permission at Uppsala University (Uppsala, Sweden). Data access in ULSAM is granted through the Interdisciplinary Collaboration Team on Uppsala Longitudinal Studies (ICTUS; https://www2.pubcare.uu.se/ULSAM/res/proposal.htm; contact: vilmantas.giedraitis@pubcare.uu.se). Data from the PIVUS study are available from the PIVUS steering committee (https://www.medsci.uu.se/pivus/; contact: lars.lind@medsci.uu.se). De-identified raw mass spectrometry data (without phenotype or other identifying information) can be obtained without prior approval from the main author (christoph.nowak@ki.se), as can all analysis code in R.
