# Supplementary material for: Non-targeted urine metabolomics and associations with prevalent and incident type 2 diabetes
Source: Sci Rep. 2020 Oct 5;10:16474. doi: 10.1038/s41598-020-72456-y (PMC7536211; doi:10.1038/s41598-020-72456-y)
Supplement: Supplementary file 1 — Supplementary file1 [file 41598_2020_72456_MOESM1_ESM.pdf]

nature publishing group npg

In accordance to Nature Publishing Groups Authorship Policy we agree to change the authors of the manuscript as indicated below.

NAME OF JOURNAL: Scientific Reports

TITLE OF MANUSCRIPT: Non-targeted urine metabolomics and associations with prevalent and incident type 2 diabetes

MANUSCRIPT NUMBER: doi: 10.1038/s41598-020-72456-y

CORRESPONDING AUTHORS NAME: Christoph Nowak

PREVIOUS AUTHOR NAMES:
1. Christoph Nowak
2. Samira Salihovic
3. Corey D. Broeckling
4. Andrea Ganna
5. Jessica E. Prenni
6. Johan Sundström
7. Christian Berne
8. Lars Lind
9. Erik Ingelsson
10. Tove Fall
11. Johan Ärnlöv

UPDATED AUTHOR NAMES:
1. Samira Salihovic
2. Corey D. Broeckling
3. Andrea Ganna
4. Jessica E. Prenni
5. Johan Sundström
6. Christian Berne
7. Lars Lind
8. Erik Ingelsson
9. Tove Fall
10. Johan Ärnlöv
11. Christoph Nowak

CHANGE TO AUTHOR LIST:
1. Samira Salihovic
2. Corey D. Broeckling
3. Andrea Ganna
4. Jessica E. Prenni
5. Johan Sundström
6. Christian Berne
7. Lars Lind
8. Erik Ingelsson
9. Tove Fall
10. Johan Ärnlöv

11. Christoph Nowak

Print Name	Signature	Date
1. Samira Salihovic		September 8, 2020
2. Corey D. Broeckling		2020-09-08
3. Andrea Ganna		2020-09-08
4. Jessica E. Prenni		09/08/2020
5. Johan Sundström		2020-09-08
6. Christian Berne		September 8, 2020
7. Lars Lind		September 8, 2020
8. Erik Ingelsson		September 8, 2020
9. Tove Fall		Sept 8th 2020
10. Johan Ärnlöv		September 8, 2020
11. Christoph Nowak		8 Sept 2020
